# Application of Reverse Genetics in Functional Genomics of Potyvirus

**DOI:** 10.3390/v12080803

**Published:** 2020-07-26

**Authors:** Maathavi Kannan, Zamri Zainal, Ismanizan Ismail, Syarul Nataqain Baharum, Hamidun Bunawan

**Affiliations:** 1 Institute of Systems Biology, Universiti Kebangsaan Malaysia, Bangi 43600, Malaysia; maathavikannan95@gmail.com (M.K.); zz@ukm.edu.my (Z.Z.); maniz@ukm.edu.my (I.I.); nataqain@ukm.edu.my (S.N.B.); 2 Department of Biological Sciences and Biotechnology, Faculty of Science and Technology, University Kebangsaan Malaysia, Bangi 43600, Malaysia

**Keywords:** potyvirus, reverse genetics, virus biology, transmission, viral protein, virus–host interaction, viral vector

## Abstract

Numerous potyvirus studies, including virus biology, transmission, viral protein function, as well as virus–host interaction, have greatly benefited from the utilization of reverse genetic techniques. Reverse genetics of RNA viruses refers to the manipulation of viral genomes, transfection of the modified cDNAs into cells, and the production of live infectious progenies, either wild-type or mutated. Reverse genetic technology provides an opportunity of developing potyviruses into vectors for improving agronomic traits in plants, as a reporter system for tracking virus infection in hosts or a production system for target proteins. Therefore, this review provides an overview on the breakthroughs achieved in potyvirus research through the implementation of reverse genetic systems.

## 1. Introduction

Reverse genetics of RNA viruses refers to the generation of recombinant viruses through site-directed mutagenesis such as substitution, deletion or insertion [1]. This consequently made various phenotypic studies possible, apart from providing a powerful tool to enhance our knowledge on life cycles and pathogenic mechanisms of RNA viruses as well as the structure or function of individual viral genes [2,3]. Besides that, reverse genetics approaches have largely contributed to the development of antiviral therapeutics, vaccines [4,5], and vectors [6]. The first success in reverse genetics for an RNA virus was reported for poliovirus, a positive-stranded RNA virus in 1981 [7]. Since then, reverse genetics technology has greatly revolutionized the studies on almost every group of positive-strand viruses including potyviruses [8].

*Potyvirus* is a genus of virus that belongs to the family Potyviridae. Potyviruses are among the most economically important and widely spread groups of plant viruses [9]. The genus comprises 158 species including *Potato virus Y* as the type species [10]. The picornavirus-like supergroup [11] is transmitted non-persistently by aphids. However, some potyviruses are also transmitted through seeds [12]. Potyviruses have a single-stranded RNA genome of around 10 kb, with a 3′ terminal poly(A) tail and a VPg protein at its 5′ end [13]. The members of potyvirus group exist as flexuous rods ranging from 720 to 900 nm in length [14]. The single open reading frame (ORF) in the RNA molecule is translated into a large polyprotein. Proteolytic cleavage of the polyprotein by three viral proteinases [15,16] gives rise to ten functional proteins as follows: P1, HC-Pro, P3, 6K1, CI, 6K2, VPg, NIa-Pro, NIb, and CP [17,18]. Additionally, the expression of a P3N-PIPO protein has been recently exhibited as a result of either a transcriptional slippage or ribosomal frameshift [19,20].

Infectious clone technology represents the most commonly applied reverse genetics system. Cloning of an infectious clone was first described for *Brome mosaic virus* [21]. Since then, infectious clones for many other plant RNA viruses were successfully obtained as either in vivo or in vitro trancripts. For in vitro strategy, the viral cDNAs are cloned under bacteriophage promoters such as T7, T3, or Sp6 followed by the generation of in vitro transcripts [22,23]. On the other hand, in vivo infectious transcripts are driven by *Cauliflower mosaic virus* 35S promoter in a binary vector. Cells transformed with the plasmids containing virus cDNA clones are then introduced into plants through agroinfiltration, particle bombardment, or rubbing onto the leaf’s surface [24,25]. In this review, we describe and summarise the application of reverse genetics in potyviral studies based on the potyvirus infectious clones developed to date.

## 2. Applications/Impacts

### 2.1. Point Mutation

Point mutation refers to a change occurred within a gene due to a single base pair alteration in the DNA sequence. During translation, conversion of RNA copied from the DNA into a sequence of amino acids would take place and point mutations usually cause a variety of effects on the protein synthesized at the final stage [26].

#### 2.1.1. Effect of Point Mutation on Virus Biological Properties

Mutagenesis studies aimed to characterize the phenotypic consequences resulting from genome modification, based on how the progenies differ from their respective wild type [27]. To date, various site-specific mutations in the potyviral genomes and screening of the progeny viruses have been conducted (Table 1). For instance, a 10-fold reduction in virus accumulation could be observed when a single point substitution, serine to glycine at position 7 was introduced in the DAS motif of CP N terminus of B11 isolate of *Potato virus A* (PVA). However, aphid transmissibility of the virus was still retained [28]. Further information on potyviral noncoding region (NCR) roles were gathered by studying the changes observed in biological characteristics of mutants with manipulated NCR. In line with that, *Clover yellow vein virus*, pClYVV cDNA clones containing no poly(A), poly(A) with shorter A residues, or various oligonucleotide sequences downstream were constructed. All RNA progenies obtained from the pClYVV cDNA constructs had poly(A) tails with infectivity varies along the sequences introduced. This suggested the addition of poly(A) to be independent of template, yet essential to maintain the infectivity of potyviral cDNA [29]. Besides that, a deletion mutation introduced in the NCR at 5′ end of *Plum pox virus* (PPV) revealed that the region between nucleotides 39 and 145 contributes to competitive fitness of the viral population [30]. A point mutation engineered into *Turnip mosaic virus*, TuMV-pXBS7/TuMV-pXBS8 chimeric viruses have allowed the mapping of a symptom severity determinant in the 3′-terminal UTR of pXBS8-derived virus [31].

#### 2.1.2. Role of Point Mutation in Cross Protection Phenomenon

Cross protection is conferred by pre-infecting the host crops with a mild virus strain to prevent the infection by another severe or closely-related strain of that virus [40]. This strategy is a promising biological method to control plant viruses [41]. However, the availability of natural attenuated strains of plant viruses is limited [42]. Hence, point mutations were intentionally engineered into plant viruses for the development of less virulent strains that could be utilized in cross protection [43]. For example, a lysine in the IDEKK motif and glycine in the HC-Pro C terminus of *Papaya ringspot virus* (PRSV) were changed to aspartic acid and lysine respectively. Consequently, PRSV mild mutants with a potential to cross-protect *Cucumis melo* plants against wild type PRSV-W were obtained [44]. Similarly, attenuated isolate M11 of *Bean yellow mosaic virus* (BYMV) was generated by exchanging an amino acid within the large ORF, leucine with serine. BYMV-M11 mutant conferred a complete cross protection against BYMV isolates from gladiolus, incomplete against BYMV isolates from other hosts, partial against a CIYVV isolate [41]. An arginine and a glutamic acid at positions, 180 and 396 in the *Zucchini yellow mosaic virus* (ZYMV) HC-Pro were substituted with isoleucine and asparagine respectively. The attenuated mutants were found to induce only mild symptoms with recovering and protected squash plants completely against the severe Taiwan strain, ZYMV TW-TN3 [42]. Another mild isolate of ZYMV, ZYMV-AG, was also generated through a substitution of isoleucine for arginine within the conserved FRNK motif of HC-Pro. Cucurbit plants inoculated with ZYMV-AG mutant were protected against the infection by severe ZYMV-NAT and ZYMV-CA isolates [45].

### 2.2. Virus/Host Interaction

Mutagenesis studies on potyvirus infectious clones allowed the mapping of viral determinants responsible for viral genome replication, local and systemic movement of virus, symptomatology and host species range [46] (Table 2). This knowledge facilitates the understanding of complex processes underlying interactions between viral and host factors during a viral infection in plants [1].

#### 2.2.1. Viral Determinants

The differential infectivity of UK 1 and JPN 1 strains of TuMV in Ethiopian mustard was studied to map the viral determinants involved [47]. Isolate UK 1 causes a systemic infection in the host while JPN 1 does not. UK 1 and JPN 1 recombinant viruses were made by exchanging the amino acids found in one isolate to that in the other isolate at several positions within the P3 C-terminal domain, leading to the identification of two adjacent positions (1099 and 1100) in TuMV-JPN 1 as the main resistance determinants. GFP-tagged viruses were also constructed to analyse the resistance of Ethiopian mustard to isolate JPN 1. Consequently, both inoculated and non-inoculated leaves showed a virus-induced fluorescence in separate areas, indicating that the non-host resistance is only apparent. In another previous study, the NIb protein of *Potato virus Y*, PVY-SON41 was demonstrated as the avirulence factor corresponding to *Pvr* resistance gene in pepper. A single substitution of adenosine nucleotide to guanosine at position 8424 in that region is sufficient for the virulence [48].

#### 2.2.2. Host Specificity

The differential responses of UK 1 and JPN 1 strains of TuMV in different hosts; brassicas (Ethiopian mustard, Indian mustard, turnip) and radish cultivars (Icicle, round red radish with a white tip (RRRWWT), Daikon) were investigated [62]. Although both infectious clones caused infection in all three brassicas, the symptoms induced were observed to be different from each other. In the case of radish, TuMV-JPN 1 infected European types (Icicle & RRRWWT) but not Japanese Daikon radish. In contrast, TuMV-UK 1 was not able to infect any radish type, showing its distinct biological properties compared to UK 1 isolate. In addition, two more isolates of TuMV, Tu-2R1 (pTuR1) and Tu-3 (pTuC) also exhibited distinct host-specific infection phenotype and symptomatology in cabbage and radish [63]. Tu-2R1 systemically infect Japanese radish, inducing mosaic symptoms apart from very mild chlorotic mottle in cabbage (*Brassica oleracea* L.). Meanwhile, Tu-3 induced systemic chlorotic and ringspot symptoms in infected cabbage but not radish. The genomic region encoding C terminus of HC-Pro, all of P3 and N terminus of 6K1 was exchanged between pTuC and pTuR1 chimeras. The only difference between pTuR1 and pTuC was identified within the amino acid sequence of P3 gene. Hence, P3 gene was suggested to be involved in the differential phenotypes caused by TuMV isolates in both hosts.

Host specificity of two different *Johnsongrass mosaic virus* (JGMV) strains, Johnsongrass infecting JGMV-Jg and Krish strain JGMV-Kr, was studied. Transcripts of JGMV-Jg full-length chimera containing coat protein sequences from JGMV-Kr were infectious in Krish resistant sorghums, thus confirming the role of JGMV-Kr coat protein in its host specificity [64]. Similarly, PRSV strain belongs to type p (PRSV P) possessed different biological characteristics compared to type W (PRSV W). Host species range of PRSV W is limited to *Chenopodiaceae* and *Cucuribitaceae* families whereas PRSV P infects plants in papaya family (*Caricaceae*) additionally [65]. Host assays using recombinant viruses generated between PRSV P-YK and PRSV W-CI in combination with site-directed mutagenesis revealed that lysine amino acid at position 27 in NIaPro acts as the host specificity determinant in PRSV for infecting papaya [66]. A single amino acid mutation, either lysine to aspartic acid or vice versa at position 27 is sufficient for the switching of PRSV host range between non-papaya infecting and papaya-infecting respectively [67].

#### 2.2.3. Virus-Host Cell Machinery Interaction

Naderpour and Johansen [68] studied *Bean common mosaic virus* (BCMV) interaction with its host, bean genotypes carrying different combinations of resistance genes (*bc-u*, *bc-1*, *bc-2*, *bc-3*, and *I*). The experimental plants were agroinoculated with an infectious clone of RU1 strain, pCA-RU1-GUS containing *UidA* gene. In situ histochemical GUS assays revealed that DW, The Prince, CRM, SP, and SGR cultivars (carrying *bc-u* resistance gene) were all systemically infected. However, the genotypes carrying the *I* gene alone (Widusa) or in combination with *bc-1* (Topcrop, ITG), *bc-12/bc-22* (IVT-7233), and *bc-3* (USCR-7, Raven) showed only a weak blue staining. These resistance responses against BCMV-RU1 are largely in agreement with previous results obtained through immunological method and symptom descriptions based analysis [69]. The study suggested that resistance gene *I* conferred a complete protection against BCMV-RU1 while *bc*-genes do not provide any. Besides that, the host defence response of cucurbit genotype, Dina-1 against ZYMV-NAA potyvirus has also been focused on previously [70]. Switches in amino terminus of the virus coat protein breaks Dina-1 resistance against ZYMV-NAA, suggesting its resistance gene product to be involved in direct interaction with the substituted region.

To learn more on mechanisms of interrelation between potyvirus and host, the impact induced by TuMV infection on the endomembranes of host early secretory pathway was examined [71]. TuMV infection caused the endoplasmic reticulum (ER), Golgi apparatus, COPII coatamers, and chloroplasts being amalgamated as a perinuclear globular structure that included viral protein, 6K_2_ vesicles too. TuMV 6K_2_ fused to photoactivable GFP (PAGFP) was used to monitor the vesicle movement. Viral egress is shown to begin with the budding of 6K_2_ vesicles at ER export site in the globular structure. The virus then travels to plasma membrane and plasmodesmata to be delivered into neighboring cells. Some peripheral vesicles were also observed to be recycled back to the globular structure. This indicated a functional linkage between the peripheral vesicles and perinuclear structure. Although the Golgi apparatus and ER lost their organization due to the amalgamation, they were still connected to the host secretory pathway (ER-to-Golgi transport). The importance of this connection was investigated by inhibiting the pathway. As a result, the disruption enhanced the clustering of peripheral 6K_2_ vesicles with COPII coatamers, leading to an inhibition of cell-to-cell movement of virus. This suggests the requirement of a functional secretory pathway for a successful intercellular propagation of TuMV.

Along with these host-viral factor interactions, the interplay between *Tobacco etch virus* (TEV) and *Arabidopsis thaliana* proteins was also investigated [72]. An affinity polypeptide Twin-Strep-tag (TST) was inserted between codons of VPg and NIaPro domains in the TEV NIa to facilitate the study. A total of 232 different *Arabidopsis thaliana* proteins targeted by viral proteins were identified through affinity purification followed by mass spectrometry analysis (AP-MS). VPg and NIaPro specifically targeted 89 and 76 of these proteins, respectively. Overall, a total of 67 proteins targeted by both domains were considered to be the targets of full-length NIa.

### 2.3. Viral Proteins

Apart from the mature proteins, proteolytic processing of the potyviral polyprotein also produced multiple partially processed intermediates [73]. The different potyviral proteins act in a coordinated and interdependent manner. About 33 interactions were identified between potyviral proteins through a testing of 58 protein combinations in planta [74,75]. This broad network of interrelations with different viral and host proteins contributes to the multifunctional nature of potyviral proteins [76].

#### 2.3.1. Functional Importance

Potyviral HC-Pro and CP proteins were reported to have a crucial role in efficient aphid transmission [28]. In line with that, a change of lysine amino acid to glutamic acid at the position 307, within Zinc-finger motif of HC-Pro completely abolished insect transmission activity of *Tobacco vein mottling virus*, TVMV-WT. The mutation suggested might have altered the motif structure and thus the binding property of HC-Pro too [32]. Likewise, a TEV mutant, TEV-2del_r_ lacking 207 nucleotides in HC-Pro sequence exhibited an aphid-non-transmissible phenotype. However, its transmission activity was restored partially by pre-feeding the aphids on active HC-Pro from PVY. This confirms the helper activity of the N-terminal domain of HC-Pro [11]. A mutation introduced within CDNQLD motif of ZYMV-A HC-Pro also resulted in an almost complete absence of symptoms and partial reduction of viral accumulation [77]. The motif sequence was suggested to be involved in symptomatology or silencing inhibition. On the other hand, when glutamic acid at amino acid position 68 within the CP of PVY-N605 was substituted with a lysine, aphid transmission of the virus increased by two folds [35].

Seo et al. [78] conducted a yeast two-hybrid system (YTHS) and galactosidase assays to investigate the interaction between CP and HC-Pro in *Soybean mosaic virus*, SMV-G7H. A highly conserved histidine in the CP C-terminus and an arginine near the cleavage site at HC-Pro C-terminus were mutated and the results obtained showed that both amino acids are necessary to maintain the interaction for a successful transmission of SMV by aphids. Moreover, an amino acid substitution in the DAG motif was found to have disrupted the CP–HC-Pro interaction in YTHS.

#### 2.3.2. Structural Importance

In order to figure out the function of 3′-UTRs of potyviruses, the sequences between nucleotide positions 8–42 in the 3′ UTR of a *Tobacco vein banding mosaic virus*, TVBMV-HN39 infectious clone, pCaTVBMV-GFP were deleted. As a consequence, the mutant caused no systemic infection in inoculated *Nicotiana benthamiana* plants. According to the RNA secondary structures prediction, the deleted region is able to form a stem-loop (SL) like structure. Progenies derived from TVBMV mutants lacking nucleotides between positions 1 and 20 and 15 and 35 within 3′-UTR were found to have restored the 5′-end SL like structures and systemically infected tobacco plants. Hence, the 5′-terminal stem loop was proposed to be neccessary for TVBMV systemic infection [79]. Formation of the stem-loop structure by a conserved nucleotide motif in 3′ UTRs of 15 potyviruses including the New Zealand isolate of *Clover yellow vein virus* (CYVV-NZ) has been reported previously [80].

### 2.4. Viral Vectors

Apart from bacterial and yeast expression systems, the plant viral vectors also provide a fast and efficient approach for synthesis of specific proteins in plant cells [81]. In this context, potyviruses have often been used as gene expression vectors due to some of their advantageous traits [12]. For instance, their rod shape make them less restrictive to accommodate large genome inserts [82]. Furthermore, it is well-known that potyviruses infect all types of plant tissues including seeds [83,84,85]. Two different insertion sites in potyviruses were exploited for the introduction of target genes, either between P1 and HC-Pro or else between NIb and capsid protein cistrons [86]. These criteria allowed a simultaneous expression of two foreign proteins [87], either as free molecules or fused to viral proteins [88,89].

#### 2.4.1. Gene Tagging

Viral vectors are usually tested through an expression of well-analyzable reporter genes in plants [46]. With reference to that, a ZYMV full-length clone containing GUS gene under a *Strawberry vein banding virus* (SVBV) viral promoter was inoculated into experimental host plants [90] (Table 3). The GUS gene was found to be expressed stably in infected tobacco plants, indicating the applicability of ZYMV infectious clone as a viral vector and the functionality of the novel SVBV promoter in driving ZYMV infection. Apart from that, tagging a TEV clone with GUS marker gene eased the monitoring of virus replication and spread following infection through a simple histochemical assay in situ [91] (Table 3).

#### 2.4.2. Expression of Biologically Active Polypeptide

The potential of *Brome mosaic virus* (BMV) to be developed into a plant virus vector was successfully demonstrated in 1986 [92]. Since then, massive efforts have been taken in constructing vector systems with plant viruses for the expression of foreign genes *in planta* (Table 4). The main goal of plant genetic modifications by incorporating transgenes is to change crop properties, leading to increased yields or higher quality of the agricultural products. For instance, a soybean glutamine synthetase (GS) together with GFP were expressed using a CIYVV-vector system, resulting in glufosinate herbicide tolerance and early flowering of legume plants [84]. In a similar way, the expression of endoglucanase D (EngD) in *N. benthamiana* using a *Pepper mottle virus*, PepMov-based vector led to an increased senescence along with milder symptoms [93]. Another purpose of developing transgenic plants is to produce different foreign substances of protein nature [46]. For an example, the nucleocapsid proteins (NPs) of tospoviruses were expressed by a ZYMV vector in squash plants. Those NPs act as immunogens for the production of highly specific polyclonal antiserum and monoclonal antibody [81]. Likewise, a transcription factor, *Rosea1* tagged infectious clone of PVY was developed, conferring benefits to molecular farming by rapidly produced larger amounts of anthocyanins in biofactory crops [94].

## 3. Conclusions

Reverse genetics in virology relies on cDNA intermediates in order to genetically manipulate RNA viruses and further produce biologically active RNA molecules. Likewise, the available infectious full-length cDNA clones of potyviruses enable countless reverse genetics studies on phenotypic alteration and cross-protection by mutated viruses as well as in determining viral elements responsible for a particular biological characteristic of the virus. Apart from improving our understanding on the complexities of interactions between host and viral factors, reverse genetics strategies have also made various applications of recombinant vectors possible. Furthermore, the approach contributed immeasurably to the elucidation of basic functions of potyviral proteins, certain motifs or genome sequences in viral replication, transmission, and cell-to-cell movement. All these findings, in conjunction with different approaches such as transciptomics and proteomics analyses, would lead to the building of a larger network that helps to further explore potyviruses, especially in the identification of more durable resistance genes. Hence, reverse genetics technologies of potyviruses are believed to hold great promise for commercial applications in the future. Despite these breakthroughs, although various viral determinants have been identified through plant–potyvirus pathosystems, the host targets of those determinants are yet to be characterized in the future.

## Figures and Tables

**Table 1:** Applications of reverse genetics techniques on biological properties related studies of potyviruses.

Virus	Genome Manipulation	Findings	Reference
*Tobacco vein mottling virus* TVMV-WT	Amino acid substitution of Lys_307_→Glu_307,_ Thr-Ser_283/284_→Ile-Asp_283/284_ and Thr-Ala_368/369_→Leu-Glu_368/369_ in HC-Pro	TuMV-307, TuMV-283/284 and TuMV-368/369 mutants-infected plants had no symptoms (11–30 day after inoculation) and showed reduced virus accumulation (Week 1–4).	[32]
*Tobacco etch virus* TEV	pTEV7D-GUS contains site-directed mutagenesis in GUS-HC-Pro fusion protein: TEV-2del_r_ retained nucleotides GUS_1_→GUS_135_ but lost HC-Pro sequence up to nucleotide 207 TEV-7del_r_ retained nucleotides GUS_1_→GUS_9_ but lost HC-Pro sequence up to nucleotide 265	The sequence deleted from TEV-2del_r_ and TEV-7del_r_ composed the N-terminal domain and a cysteine-rich motif of HC-Pro. The ability of these mutants to replicate and move systemically indicated that the N-terminal domain of HC-Pro is not a factor essential for these processes. Although both TEV-2del_r_ and TEV-7del_r_ were viable in plants, a negative effect on accumulation of viral RNA and coat protein could be observed, suggesting a potential function of HC-Pro in enhancing viral replication.	[11]
*Plum pox virus* PPV-D	PPV mutant that lacked long sequences located between nucleotides 39 and 145 in 5′ NCR (146 nt long)	The deleted region is not necessary for genomic RNA replication, but contribute to the competitive fitness of the PPV since the mutants were not able to compete with the wild-type strain in co-inoculation experiments.	[30]
	PPV mutant without sequences between nucleotides 127 and 145 in 5′ NCR	Plant infected with PPV mutant viruses ∆[127,145] showed a very mild symptoms. However, the wild-type symptom severity was recovered after spontaneous second-site mutations.	
*Clover yellow vein virus* CIYVV	Mutations in poly(A) (10 A residues) of pClYVV: pClYVV-PA5: Shorter by 5 (A) pClYVV∆PA: Poly(A)-deficient	Mutants still infectious, but the infectivity was reduced compared to that of pClYVV	[29]
	pClYVV PAmut series (pClYVV PAmut 1,2,3,4) consisted of plasmids with T residues in the poly(A) tract	Mutants with more than five continuous A residues (pClYVV PA5, -PAmut 2, and -PAmut 3) are more infectious than those having zero to two A residues (pClYVV∆PA, -PAmut 1, and -PAmut 4). Hence, minimum 5–10 (A) residues required for high infectivity.	
	pClYVV 3′dup 1: Last 5 nts, CGAGA was duplicated pClYVV 3′dup 4: Last 10 nts, TAGAGCGAGA was duplicated	The infectivity of pClYVV 3′dup 4 was higher than that of pClYVV 3′dup 1. This suggested that the length of duplicated sequence (downstream of the poly(A) site) might have enhanced mutants’ infectivity.	
*Clover yellow vein virus* CIYVV	A series of pClYVVdel mutants with a deletion of various parts in 3′NCR A series ofpClYVV plasmids containing duplicated 3′ terminal region	All deletion mutants lacking any portion in 3′NCR were not infectious. Various mutants with duplicated 3′ terminal sequences were infectious only when the authentic 3′ terminal sequence was restored, probably by recombination, and none of the constructs retained the original sequence in progeny viral RNA. The 3′-terminal region of ClYVV contains *cis*-acting elements that are strictly necessary for ClYVV replication.	[33]
*Potato virus A* PVA-B11 &	Replacement of the entire CP gene of PVA-B11 with the CP gene of PVA-U (B11-U_CP_)	Virus accumulation in tobacco reduced 5-fold, to the level of PVA-U.	[28]
PVA-U	Four simultaneous amino acid substitutions (E88K, H89Y, G153S and A330T) made in PVA-B11 HC-Pro (according to PVA-U HC-Pro)	Virus accumulation in tobacco increased 2- to 4-fold, to the level of PVA-B11.	
	Simultaneous mutation of HC-Pro and replacement of CP in PVA-B11	Delayed systemic movement in tobacco and limited cell-to-cell movement in potato.	
	Amino acid substitution of S_7_→G_7_ in CP of PVA-B11	The PVA-B11 CP contains a DAS motif (aa 5–7) and is not aphid-transmissible whereas PVA-U contains a DAG motif and is aphid-transmissible. S7G mutation restored aphid transmissibility of PVA-B11.	
*Potato virus A* PVA-B11	PVA containing: GFP inserted at the junction of the NIb and CP (35S-PVA-GFPNIb/CP) All three potential CK2 phosphorylation sites in CP (Thr-242, Thr-243, and Ser-244) substituted either by non-phosphorylatable Ala/Asp/Tyr residues	Mutant viruses were defective in cell-to-cell and long distance movement, suggesting the vital regulatory role of PVA CP phosphorylation by CK2 in virus infection. However, at 20 dpi, weak GFP fluorescence appeared in the upper leaves. Reverse transcriptase–mediated PCR and nucleotide sequencing revealed a reverse mutation from Ala-243 to Thr-243, indicating that the virus had to restore at least one CK2 site to remain viable.	[34]
*Potato virus Y* PVY-N605	Amino acid changes within CP: Asn_25_→Ile_25_ Glu_68_→Lys_68_	Asn_25_Ile mutation significantly increased PVY accumulation and competitiveness in tobacco but decreased its competitiveness in potato Glu_68_Lys mutation significantly increased PVY accumulation in the absence of competition in potato but strongly decreased its competitiveness in that host	[35]
*Tobacco vein banding mosaic virus* TVBMV HN39	Amino acid substitution of R_182_→I_182_ and D_198_→K_198_ in FR182NK and CD198N motifs of HC-Pro respectively	Mutation of R182I/D198K reduced symptoms and virus accumulation of pTVBMV in inoculated *Nicotiana benthamiana* plants significantly, indicating a role of the two amino acids in regulating virulence of TVBMV.	[36]
*Soybean mosaic virus* SMV-G7d	Amino acid changes: Lys_64_→Arg_64_ in P1 Gln_472_→Arg_472_ in HC-Pro Val_823_→Met_823_, Met_915_→Val_915_, Lys_953_→Glu_953_, Ala_1112_→Val_1112_ in P3 Val_2842_→Met_2842_ in CP	Long-term passage of progenies of molecularly cloned SMV strain G7 in *Rsv1*-genotype soybean resulted in an emergence of a mutant, SMV-G7d. A total of seven amino acid substitutions in SMV-G7d genome lead to its incapability in provoking either *Rsv1*-mediated lethal systemic hypersensitive response or PR-1 protein gene transcript upregulation as parental SMV-G7 and thus evade an *R*-mediated recognition.	[37]
*Soybean mosaic virus*	Amino acid substitution of N_286_→D_286_ in HC-Pro of SMV A297-12	The change N286D reduced silencing suppressor activity of SMV A297-12.	[38]
SMV A297-12,SMV A297-13, SMV 413	Substitution of the HC-Pro in SMV 413 infectious clone with that of: A297-12 (HC-Pro_(L54, N286, D369)_) producing SMV 413-HC-12 (HC-Pro_(F54, D286, N369)_) producing SMV 413-HC-13	RNA accumulation of SMV 413-HC-13 was reduced to less than 3% of the level of SMV 413-HC-12 at 10 dpi but increased to 40% of SMV 413-HC-12 at 40 dpi. At 50 dpi RNA accumulation of SMV 413-HC-13 was similar to that of SMV 413-HC-12 and the D at position 286 of HC-Pro in SMV 413-HC-13 was found to have reverted to N, indicating the strong selection for revision to wild type when the mutation was introduced into SMV 413 infectious clone.	
*Papaya ringspot virus* PRSV-W	Null mutant pSAHPdelF1 retained first & last 5 aa while pSAHPdelF2 retained first 2 aa of HC-Pro to maintain the integrity of P1/HC-Pro proteolytic site. pSAHPdelN54 contains a deletion of first 54 aa at HC-Pro N-terminus	Both null mutants (pSAHPdelF1 & pSAHPdelF2) were non-infectious in the zucchini host. Infectivity test using pSAHPdelN54 indicates that deletion of as few as 54 amino acids at the N-terminus of HC-Pro is deleterious for PRSV systemic infection in zucchini.	[39]

**Table 2:** List of molecular determinants mapped in potyviral genomes through reverse genetics method.

Virus	Genome Manipulation	Application	Findings	Reference
*Turnip mosaic virus* TuMV	Chimera pXBS78 derived from pXBS7 contains a fragment of 8975–9311 residues from 3′ terminal UTR of pXBS8 Chimera pXBS87 derived from pXBS8 contains a fragment of 8975–9253 residues from 3′ terminal UTR of pXBS7	Genetic determinant (symptom severity)	pXBS7 induced symptoms in infected tobacco plants that are indistinguishable from those produced by native TVMV RNA. In contrast, pXBS78 induced only very mild barely detectable symptoms in infected plants. The results of sequence analysis and genome exchange experiments indicate that a 58-nt segment consisting of patterns of adenine and uracil residues in the 3′ UTR of pXBS8-derived RNA is responsible for the symptom attenuation phenotype.	[31]
*Pea seed-borne mosaic virus* PSbMV-P1 & PSbMV-P4	Exchange of 5′UTR, P1pro and HCpro of P-1 with the corresponding regions from P-4 creating: vP-1(P-4 5′UTR) vP-1(P-4 P1pro) vP-1(P-4 HCpro) respectively	Genetic determinant (seed transmission)	P-1 is highly seed-transmitted whereas P-4 is rarely seed-transmitted. The seed transmission frequencies of vP-1(P-4 5′UTR) and vP-1(P-4 HCpro) were reduced to 50% and 20% of vP-1, respectively, while vP-1(P-4 Plpro) was seed transmitted at the same frequency as vP-1. This showed that the HC-Pro was a major determinant of seed transmission while the P1pro showed no measurable influence.	[49]
*Tobacco etch virus* TEV HAT & TEV NW	TEV HAT and TEV NW chimeras with exchanged coding regions of the P3, the CI, and the 6-kDa and VPg-NIa proteins	Genetic determinant (wilting response)	TEV HAT causes wilting in Tabasco pepper whereas TEV NW is a non-wilting strain. TEV HAT possessed two wilting determinants: 3′ one third of the P3 coding region 3′ end of the CI, the 6-kDa protein and the 5′ end of the VPg-NIa coding regions Replacement of only one of them with its counterpart from TEV NW does not alter the wilt phenotype.	[50]
*Plum pox virus* PPV-R & PPV-PS	Chimera containing 3109–3628 coding region (173 aa) from PPV-PS on PPV-R background	Genetic determinant (symptom development)	The region encodes for C-terminal part of P3+6K_1_, differ at 11 positions between PPV-R and PPV-PS and contains all information required to transform the R-type into PS-type symptomatology in *Nicotiana clevelandii*.	[13]
*Potato virus Y* PVY-N, PVY-NTN & PVY-O	PVY-N/NTN and PVY-N/O chimeras carry the 3′ end of NIb, the whole CP and 3′UTR region of PVY^NTN^ and PVY^O^, respectively, in a PVY^N^ genetic background	Genetic determinant (symptom development)	In five (*N. benthamiana*, *N. tabacum*, *N. glutinosa*, *Solanum tuberosum*) of the six hosts, the chimeras induced similar symptoms to those of PVY^N^. By contrast, in *Physalis floridana*, the N/O hybrid caused symptoms similar to those of the 3′NIb-CP-donating PVY^O^ strain, thus suggesting symptom determinants to be different even between strains of the same virus species in a particular host.	[51]
*Potato virus Y* PVY^N^-605 & PVY^O^-139	Chimeric PVY^N/O^ isolates by replacement of selected PVY^N^ sequences with sequences from PVY^O^-139 on N-605 background using restriction sites: PVY^N/O^NrBg: [*Nru*I(2086)-*Bgl*II(2763)] PVY^N/O^NrBz: [*Nru*I(2086)-*Bst*Z17I(2591)] PVY^N/O^NrSp: [*Nru*I(2086)-*Spe*I(2257)] PVY^N/O^SpBz: [*Spe*I(2257)-*Bst*Z17I(2591)]	Genetic determinant (symptom development)	PVY^N^ induce veinal necrosis on *N. tabacum* leaves whereas PVY^O^ isolates induce only mottling and mosaic symptoms on tobacco. The inability of PVY^N/O^NrBg and PVY^N/O^NrBz to induce necrosis on *N. tabacum* shows the significance of the PVY^N^-605 region (nt 2087–2591) in inducing tobacco vein necrosis (TVN) symptom. Sequence comparison between five PVY^N^ and three PVY^O^ isolates in this region revealed that only the HC-Pro residues at locations 400 (nt 2212–2214) and 419 (nt 2269–2271) were restricted to necrotic (K400 and E/G419) or non-necrotic (R400 and D419) isolates. The *Spe*I site used in the PVY^N/O^NrSp and PVY^N/O^SpBz chimeras located between K400 and E419 codons. Both mutants were unable to induce necrosis symptoms on *N. tabacum*, indicating the requirement of K400 and E419 in the HC-Pro sequence for TVN process.	[52]
*Potato virus Y* PVY^N^-605 & PVY^O^-139	Amino acid substitution of N_339_ in PVY^N^-605 with sequence (D_339_) from PVY^O^-139	Genetic determinant (symptom development)	PVY^N^ induce veinal necrosis on *N. tabacum* leaves whereas PVY^O^ isolates induce only mottling and mosaic symptoms on tobacco. N_339_D mutant resulted in the modification of the PVY^N^ biological property, from necrotic to mosaic symptoms on infected *N. tabacum*.	[53]
*Pea seed-borne mosaic virus* PSbMV-DPD1 & PSbMV-NY	DPD1(CP-NY) and NY(CP-DPD1) chimeras with exchanged CP p35S-NY(S47P) carries amino acid substitution of Ser_47_ in NY CP with Pro_47_ as in DPD1 CP and vice versa in p35S-DPD1(P47S)	Genetic determinant (long-distance movement)	DPD1 spreads to uninoculated leaves, whereas NY is restricted to the inoculated leaves. DPD1(CP-NY) was restricted to inoculated leaves whereas NY(CP-DPD1) infected *C. quinoa* systemically. Hence, CP was identified as the determinant of long-distance movement. Ser_47_→Pro_47_ permit systemic spread of the NY(S47P) mutant whereas Pro_47_→Ser_47_ restricted DPD1(P47S) reverse mutant to inoculated leaves.	[54]
*Turnip mosaic virus* TuMV-UK1	Nucleotide substitution of A_5056_→G_5056_ (p35Tunos 5056A>G) and A_5570_→G_5570_ (p35Tunos 5570A>G) in the CI coding region of p35Tunos	Pathogenicity determinant	TuMV UK 1 is incapable of infecting lines R4 (possessing the resistance gene *TuRB01*). Unlike the wild-type TuMV UK 1 derived from p35Tunos, recombinant viruses containing the mutations infected the line N-o-1 (possessing *TuRB01*), confirming that either single nucleotide change is sufficient to overcome *TuRB01* resistance.	[55]
*Lettuce mosaic virus* LMV-0 & LMV-E	LMV-0 and LMV-E chimeras with exchanged HC-Pro and 3′ half coding regions	Pathogenicity determinant	LMV-E could overcome the protection afforded by the resistance genes *mo1*^1^ or *mo1*^2^, resulting in systemic mosaic symptoms whereas LMV-0 could not. HC-Pro of LMV-E is responsible for causing severe stunting and necrotic mosaic in susceptible cultivars. In contrast, the ability to overcome *mo1* resistance was mapped to the 3′ half of the LMV-E genome.	[56]
*Turnip mosaic virus* TuMV-UK1	Mutation at position +3394 (P3 sequence) and +5447 (CI coding region) in TuMV-UK 1M3	Pathogenicity determinant	Mutation in the P3 protein defeated immunity conferred by resistance gene *TuRB04. TuRB04* is epistatic to the second gene, *TuRB05*. Mutation in the CI breaks *TuRB05* resistance in *Brassica napus* differential line 165.	[57]
*Pea seed-borne mosaic virus* PSbMV-P1 & PSbMV-P4	PSbMV-P1 and PSbMV-P4 chimeras with exchanged VPg coding region	Virulence determinant	PSbMV-P4 is fully infectious in the *sbm-1/sbm-1* genotype whereas PSbMV-P1 is not. P-1/P-4 recombinant clones with the VPg (21-kDa) from P-4 was capable in overcoming *sbm-1* resistance, while chimeric clones containing the P-1 VPg domain were not infectious to *sbm-1/sbm-1* peas. VPg acts as the PSbMV determinant of infectivity in *sbm-1/sbm-1* peas.	[58]
*Potato virus Y* PVY-LYE84.2, PVY-LYE84, PVY-SON41	Chimera LYE84.2×VPgLYE84: central part of VPg (Nt 5762–6129) in PVY-LYE84.2 exhanged with respective region from PVY-LYE84 LYE84.2×VPgSON41 & SON41×VPgLYE84.2 chimeras with exchanged central part of VPg (Nt 5923–6118)	Virulence determinant	SON41×VPgLYE84.2 chimera and PVY-LYE84.2 systemically infected PI247087, *Lycopersicon hirsutum* carrying *pot-1* resistance gene while LYE84.2×VPgLYE84 does not. Difference identified within the exchanged region between both isolates is that, LYE84.2 contains Arg at amino acid position 119 whereas LYE84 contains His instead. Hence, Arg_119_ is responsible for the virulence toward *pot-1*.	[59]
			Chimera LYE84.2×VPgSON41 and PVY-SON41 systemically infected all three *Capsicum annuum* genotypes including Yolo Y and Florida VR2 carrying *pot-pvr2^1^ & pvr2^2^* resistance genes while SON41xVPgLYE84.2 infected Yolo Wonder, susceptible cultivar only. SON41 and LYE84.2 differ by five amino acids within the exchanged region (at positions 105, 115, 119, 121, 123) which suggested to affect the virulence towards *pvr2^1^ & pvr2^2^* genes.	
*Potato virus Y* PVY-SON41p	Amino acid substitution of D_119_→N_119_ & H_121_→N_121_ in VPg cistron	Virulence determinant	Virulence of PVY towards an allelic series (*pvr2^1^*, *pvr2^2^*, *pvr2^3^* resistance alleles) at the *pvr2* locus in pepper genotypes are related to variations in the VPg. PVY isolates; To72, CAA82 and EP03 (H_119_, N_121_) displayed the closest VPg sequence compared to SON41p (D_119_, H_121_) and its mutants virulent towards *pvr2^2^*, differing by two amino acids. Selective pressures by the *pvr2^1^* and *pvr2^3^* alleles accelerate the fixation of the first mutations required for virulence towards *pvr2^2^*. The use of *pvr2^1^* or *pvr2^3^* could contribute to selection for the reverse mutant D_119_N (by fixing the N_121_H mutation), while *pvr2^3^* could select for the reverse mutation H_121_N (by fixing the N_119_D mutation). These mutants then need only single-nucleotide substitution for virulence against *pvr2^2^*.	[60]
*Potato virus Y* PVY-SON41	Nucleotide substitution of A_8424_→G_8424_ in NIb cistron of SON41p	Virulence determinant	NIb protein of PVY was the avirulence factor corresponding to resistance gene *Pvr4*. However, A_8424_→G_8424_ substitution was sufficient for virulence and imposed a high competitiveness cost to the virus against an avirulent PVY variant in plants lack of *Pvr4.* The only observed possibility of the virulent mutant to increase its fitness was through the G_8424_A reversion, which simultaneously led to a reversion of the virus virulence, strengthening the high durability potential of the *Pvr4* resistance in field.	[48]
*Turnip mosaic virus* TuMV-UK1 & TuMV-JPN 1	U(2511-3767)J+GFP chimera with 2511-3767 (P3 protein) from JPN1 in UK1 background UK 1 and JPN 1 chimeras with exchanged 1099–1100 amino acids	Resistance determinant	TuMV-UK 1 able to infect Ethiopian mustard while isolate JPN 1 was not. GFP tagged viruses showed that Ethiopian mustard conferred an apparent non-host resistance (NHR) to JPN 1, as virus-induced fluorescence could be found in discrete areas of both inoculated and non-inoculated leaves. Two adjacent positions (1099 & 1100) in the C-terminal domain of P3 were identified as the resistance determinants in TuMV-JPN 1.	[47]
*Turnip mosaic virus* TuMV-UK1 & TuMV-CDN 1	UK 1 *P3* I153F carries amino acid substitution of Ile_153_ in P3 with Phe_153_ as in CDN 1 and vice versa in CDN 1 *P3* F153I	Avirulence determinant	The resistance gene *TuRB03*, in the *B. napus* line 22S, is effective against CDN 1 but not UK 1. Virus derived from UK 1 *P3* I153F completely prevented any viral infection, affecting the phenotype of UK 1 on *B. napus* line 22S. Virus derived from CDN 1 *P3* F153I was virulent and infected line 22S with systemic mosaic symptoms. This indicate the role of P3 residue at position 153 in avirulence.	[61]

**Table 3:** Potential of potyviruses as expression vectors for the monitoring of viral infections in host plants.

Virus	Genome Manipulation	Findings	Reference
Zucchini yellow mosaic virus ZYMV	Insertion of the ∆SVBV-promoter (328 bp fragment) before the GUS reporter gene replacing 35S promoter in the binary p301, give rise to ∆SVBV-GUS-p301	∆SVBV-promoter [conserved CCACT (at -83) and TATA (at -31) boxes] from Strawberry vein banding caulimovirus (SVBV) genome, was identified as a novel putative promoter due to its ability in driving infection of the full-length ZYMV cDNA. Stable expression of GUS under the ∆SVBV-promoter was shown in transformed tobacco shoots in roots, leaves and stems.	[90]
Turnip mosaic virus TuMV-UK1	p35Tunos/nGFP–cGUS and p35Tunos/nGUS–cGFP contain reporter genes, uidA and gfp inserted in between P1 and HC-Pro/Pol and CP cistrons respectively, and vice versa	Attenuated systemic symptoms were observed in transfected Brassica perviridis and Western blot analyses showed that both foreign proteins were produced. GFP was stable over 30 days post-transfection (dpt) while uidA was gradually lost at 15 dpt at either sites. This indicate the possibility to produce two foreign proteins simultaneously in a TuMV-based vector.	[86]
Turnip mosaic virus TuMV	Plasmids vec01-GUS and vec01-GFP contain reporter genes, uidA and gfp inserted in between NIb and CP genes	Both TuMV clones were infectious in Arabidopsis, with characteristics (infectivity and symptomatology) similar to the wild-type virus.	[12]
Tobacco vein banding mosaic virus TVBMV HN39	pTVBMV-GFP contains an Aequoria victoriae gfp gene inserted between the NIb and CP encoding regions	pTVBMV-GFP expressed stably in the systemically infected N. benthamiana leaves, indicating suitability of pTVBMV as an expression vector	[36]
Papaya ringspot virus PRSV-Hainan	PRSV-GFP contains a gfp gene between the NIb and CP encoding regions	PRSV-GFP transformed into Rhizobium radiobacter caused typical symptoms and green fluorescence in inoculated papaya plants, indicating that GFP can be expressed stably in PRSV vector without affecting virus infection and movement.	[95]
Papaya ringspot virus PRSV-W	pCamPRSV-W-GFP contains a gfp gene inserted into NIb- and CP-coding region	Appearance of strong green fluorescence in systemic leaves of agro-inoculated Cucurbita pepo, Cucumis melo, Citrullus lanatus and Cucumis sativus plants indicated that pCamPRSV-W can express foreign genes effectively.	[44]
Turnip mosaic virus TuMV-YC5	In addition to the N-terminal (NT) of HC-Pro, the NT regions of P3, CIP, NIb, and CP of TuMV-YC5 were engineered for a GFP/Der p 5 ORF insertion	In addition to the NT regions of HC-Pro and CP, the NT regions of P3, CIP and NIb were also able to carry both heterologous ORFs to be translated as a part of the polyprotein and processed as free-form protein although showed more permissiveness to the GFP ORF than Der p 5 ORF. The efficiency and stability of expression of the ORFs depends on the particular ORF and the host plant employed.	[96]
Tobacco etch virus TEV	A series of six histidines (his-tag) inserted near the 5′ terminus of the HC coding region in pTEV-HCHXa	pTEV-HCHXa was infectious, produced symptoms in tobacco similarly as wild-type TEV, and stably maintained through at least 4 cycles of aphid transmission. HC protein purification based on the affinity of its his-tag for Ni2+-charged resin, yielded large amount of fully functional his-tagged HC protein.	[97]
Clover yellow vein virus CIYVV	pClYVV-GFP contains a gfp gene inserted between P1 and HC-Pro. Junctions between the inserted proteins contained the protease cleavage recognition sites	Green fluorescence was detected in broad bean, kidney bean, and soybean plants infected with pClYVV-GFP. The stability of the construct in the symptomatic tissues was confirmed by RT-PCR and Western blot analyses.	[84]
Plum pox virus PPV-D	pICPPV-NK-GFP contains a gfp gene inserted between the NIb and CP junction	GFP was detected in crude extracts from PPV-NK-GFP infected leaves by Western blot. Genetic stability of the chimera was confirmed by IC-PCR amplification of a cDNA fragment including the foreign sequence of expected size. Virus and GFP accumulations were quantified in infected N. clevelandii plants by ELISA.	[88]
Soybean mosaic virus SMV-G7H	GFP cloned into pSMV-MCS between the P1 and HC-Pro cistrons (pSMV-GFP)	Typical mild mosaic symptoms and systemic expression of GFP protein were detected in pSMV-GFP infected soybean. The GFP gene was shown to be maintained stably in soybeans even after three serial passages.	[87]
Tobacco etch virus TEV	pTEV7D contains a β glucuronidase (GUS) gene between the polyprotein-coding sequences for N-terminal 35-kDa proteinase and HC-Pro	GUS act as a marker gene in TEV genome, demonstrating that virus replication and movement can be monitored easily by using a simple histochemical assay in situ. The GUS enzyme was proteolytically excised as a fusion product with HC-Pro.	[91]
Lettuce mosaic virus LMV-E	pLMVE-GFP and pLMVE-GUS contains a jellyfish GFP & β glucuronidase (GUS) gene respectively, fused to HC-Pro	Both GFP- and GUS-tagged viruses induced attenuated symptoms in susceptible lettuce cultivars Trocadero and Vanguard, compared to wild-type. Accumulation of the recombinant viruses was either undetectable (pLMVE-GUS) or strongly delayed and inhibited by 90% (pLMVE-GFP). In contrast to parental virus, the recombinants were unable to overcome the resistance gene, mo12.	[83]
*Pepper mottle virus* PepMoV-Vb1	SP6PepMoV-Vb1/GFP contains *turboGFP* inserted between NIb and CP coding regions	Expression of GFP was monitored under illumination. SP6PepMoV-Vb1/GFP was highly infectious and symptoms were not different from those induced by either pSP6PepMoV-Vb1/wild-type PepMoV-Vb.	[98]
*Papaya leaf distortion mosaic virus* PLDMV-DF	pPLDMV-GFP and pPLDMV-mCherry contain a *GFP* and *mCherry* into the NIb/CP junction respectively	PLDMV-GFP or PLDMV-mCherry developed typical systemic symptoms in 95% of infected papaya seedlings, in which fluorescence was observed in leaves, stems, and roots. Both clones were stable in papaya for more than 90 days and during six serial passages at 30-day intervals.	[99]
*Pepper mottle virus* PepMoV	pPepMoV-I: GFP (with intron 2 of ST-LS1) contains a *gfp* gene inserted between P1 and HC-Pro	The consistent enhancement of PepMoV RNA level and translation products (GFP) observed in the study suggested a hypothesis that the intron ST-LS1 enhanced the stability and translational efficiency of the PepMoV transcripts in the infiltrated leaves.	[100]
*Plum pox virus* PPV-NAT	pPPV-H6K1-NAT contains a histidine tag inserted in the protein of a 6 kDa (6K1) coding region	For detection of 6K1 as a mature protein of 6 kDa in vivo, pPPV-H6K1-NAT enabled the concentration and purification of histidine-tagged 6K1 from infected *Nicotiana benthamiana* leaves at 4, 7 and 14 days post-inoculation (d.p.i.) through affinity chromatography.	[101]
*Plum pox virus* PPV-Rec	pIC-PPV-Rec-P1His contains a sequence coding for six histidine residues inserted between the 4th and 5th amino acid of the P1 protein	The pIC-PPV-Rec-P1His was able to replicate in *N. benthamiana* and remained stable during several mechanical passages of the virus. Immunoblot analysis with the anti-his antibody showed a diffuse band corresponding to the molecular weight about 70–80 kDa in the root samples from early stage of infection. However, this signal culminated on the sixth day post inoculation, later it rapidly disappeared.	[102]
*Potato virus Y* PVY-RB	pGPVY-Ros1 contains a *Antirrhinum majus* Rosea1 transcription factor inserted between the NIb and CP cistrons	Mechanically inoculated solanaceous plants induced the formation of red infection foci in inoculated tissue and solid dark red pigmentation in systemically infected tissue, which allows disease progression to be easily monitored Facilitated the novel quantitative analysis of antiviral activity in plants by using silver nanoparticles, a nanomaterial with exciting antimicrobial properties Enabled the visual monitoring the virus transmission by an aphid vector	[94]

**Table 4:** Applications of potyviral vectors for the expression of biologically active polypeptides.

Virus	Genome Manipulation	Application	Findings	Reference
*Clover yellow vein virus* CIYVV	pClYVV-GFP-GS contains a *gfp* gene and soybean glutamine synthetase (GS) inserted between P1 and HC-Pro. Junctions between the inserted proteins contained the protease cleavage recognition sites	Enhance crop quality	Western blot analyses showed that GFP and GS have been precisely excised from the viral polyprotein with the viral proteases (P1 and NIa). Co-ordinate expression of multiple genes can be achieved by proteolytic cleavage of a polyprotein. The plants expressing GS and GFP became tolerant to the herbicide glufosinate, and flowered early	[84]
*Plum pox virus* PPV-D	pICPPV-NK-VP60 contains the VP06 structural protein of *Rabbit hemorrhagic disease virus* (RHDV) inserted between the NIb and CP	Expression of antigen	Immunization of the natural host of RHDV, rabbits with extracts of *Nicotiana clevelandii* plants infected with the PPV-NK-VP60 chimera induced an efficient immune response that protected animals against a lethal challenge with RHDV.	[88]
*Zucchini yellow mosaic virus* ZYMV TW-TN3	ZWBNV-N recombinant contains nucleocapsid protein (NP) ORF of *Watermelon bud necrosis virus* inserted between the P1 and HC-Pro.	Expression of antigen	Six histidine residues and an NIa protease cleavage site were added at the C-terminal region of the inserts to facilitate purification and process of free form of the expressed NPs, respectively. The ZYMV-expressed WBNV NP was purified from extracts of the infected squash plants and was used as an immunogen for production of specific antiserum in a rabbit and monoclonal antibodies in mice.	[81]
*Papaya ringspot virus* PRSV-W	pVD2EDIII contains a histidine tagged dengue E protein domain III (DENV 2 E) inserted between the P1 and HC-Pro	Expression of antigen	The construct was designed to generate a discrete antigen moiety (D2EDIII) after proteolytic processing. However, the E protein insert was fused to the PRSV P1 protein, suggesting inefficient protease processing at the P1/D2EDIII junction. Despite the failure, the insert was shown to be stable over 2 passages PRSV indicating the vector suitability and stability for the expression of heterologous proteins in zucchini plants.	[103]
*Potato virus A* PVA-B11	PVA vectors containing soluble resistance-related calcium-binding protein (sorcin) catechol-O-methyltransferase (S-COMT) between NIb and CP	Expression of genes human origin	The inserts caused no adverse effects on viral infectivity and virulence, and the inserted sequences remained intact in progeny viruses in the systemically infected leaves. S-COMT with high levels of enzymatic activity were produced. However, no sorcin was detected despite the expected equimolar amounts of the foreign and viral proteins being expressed as a polyprotein.	[104]
*Zucchini yellow mosaic virus* ZYMV-AG	Non-pathogenic vector, ZYMV-AGII carries CMV coat protein (AGII-CMV-CP), jellyfish GFP (AGII-GFP), *uidA* (AGII-GUS) & human interferon-alpha 2 (AGII-IFN) genes inserted into NIb-CP site	Viral coat protein & human anti-viral drug	All four constructs infected squash plants and stably expressed the inserted genes without affecting plant development. ZYMV-AG vector could mediate the synthesis of a biologically active IFN in edible cucurbit fruit and leaves.	[105]
*Zucchini yellow mosaic virus* ZYMV	p35ZYMVDerp5 contains *Dermatophagoides pteronyssinus* group 5 allergen (Der p 5) inserted in between P1 and HC-Pro coding regions	Expression of mite allergen	Infectivity assays and immunoblotting revealed that large quantities of free-form virus-expressed Der p 5 (vDer p 5) are produced in the recombinant virus-infected squash plants. Female mice were orally treated with the vDer p 5 extract. As a result, the allergen inhibited Der p 5-specific IgE synthesis and airway inflammation, clinically relevant to human asthma. This provides a novel approach for the therapy of allergic asthma.	[106]
*Soybean mosaic virus* SMV-G7H	RNA silencing suppressors 2b and p19 genes were cloned into pSMV-MCS between the P1 and HC-Pro cistrons (pSMV-2b and pSMV-p19 respectively)	Expression of RNA silencing suppressors	Severe symptoms including stunting, extensive leaf deformation and shrivelling was detected in either pSMV-2b or pSMV-p19 infected soybean, with the accumulation of SMV RNAs and CP similar to that in plants infected with pSMV-GFP.	[87]
	Partial forward sequence (567 bp) and partial reverse sequence (282 bp) of were inserted into pSMV-MCS between the P1 and HC-Pro cistrons (pSMV-spPDSfw and pSMV-spPDSrv respectively)	Expession of phytoene desaturase (PDS)	Successful infection of both clones and stable insertion of the partial PDS genes within viral genomes were shown. However, there is no photobleached leaves in plants infected with pSMVspPDSfw/pSMV-spPDSrv could be observed, indicating that the PDS gene was not successfully silenced by the clones. This was expected since SMV encoded a strong silencing suppressor, HC-Pro.	
*Potato virus Y* PVY-RB	pGPVY-Ros1 contains a *Antirrhinum majus* Rosea1 transcription factor between NIb & CP	Regulatory factor	PVY-Ros1 induced the accumulation of of antioxidant anthocyanins (275 mg per 100 g of fresh weight) in biofactory plants in only 12 days.	[94]
*Pepper mottle virus* PepMoV-Vb1	pSP6PepMoVVb1/EngD contains an endoglucanase D (EngD) from *Clostridium cellulovorans* inserted between sequences encoding NIb and CP	Expression of enzyme	*N. benthamiana* infected with pSP6PepMoVVb1/EngD showed increased senescence but milder symptoms than wild-type PepMoV-Vb1. Glucose assay confirmed the EngD enzymatic activity in infected plants and thus, suggested the use of a viral vector for heterologous *engD* expression leading to the digestion of cellulose substrate in plant cells for biomass production.	[93]
